# Preparation and Evaluation of a Polyimide-Coated Ultrafine Gilt Molybdenum Wire and Its Knitted Mesh Used for Electromagnetic Reflectors

**DOI:** 10.3390/polym9120734

**Published:** 2017-12-20

**Authors:** Huiqi Shao, Nanliang Chen, Shuang Li, Fangbing Lin, Jinhua Jiang, Xiaofei Ma

**Affiliations:** 1 State Key Laboratory for Modification of Chemical Fibers and Polymer Materials, Donghua University, Shanghai 201620, China; 1142002@mail.dhu.edu.cn (H.S.); nlch@dhu.edu.cn (N.C.); ls@dhu.edu.cn (S.L.); 2140024@mail.edu.cn (F.L.); 2 College of Textiles, Donghua University, Shanghai 201620, China; 3 Key Laboratory of High Performance fibers & products, Ministry of Education, Donghua University, Shanghai 201620, China; 4 Xi’an Institute of Space Radio Technology; Xi’an 710100, China; maxf@cast504.com (X.M.)

**Keywords:** polyimide coating, gilt molybdenum, wire mesh, temperature stability, contact resistance stability, reflection performance

## Abstract

In this work, polyimide (PI) was coated onto an ultrafine gilt molybdenum wire in order to protect the gilt surface and prepare an electrically stable wire mesh material which can be widely used in space. The surface of the PI-coated gilt molybdenum wires was characterized using FTIR, SEM, and EDS. Factors such as temperature stability of the PI coating, mechanical properties of the PI-coated gilt molybdenum wires, contact resistance stability, and electromagnetic microwave reflectivity of the their knitted meshes were also investigated. The results indicate that the PI coating conformed uniformly to the surface of the gilt molybdenum wires. The prepared PI coating exhibited excellent temperature stability in the −196 to 300 °C range and could efficiently protect the gilt surface and improve the stability of contact resistance, while the reflection of its wire meshes showed only a slight decrease of 1.4% with the PI coating thickness of 3 μm for electromagnetic microwaves in the S band.

## 1. Introduction

Materials used in the advanced aerospace applications, such as antenna components, have to be capable of withstanding oxidation and radiation, while retaining satisfactory mechanical properties under an extremely complex environment. Molybdenum is one of the most popular metallic materials used in aerospace. Once drawn into fine wires, molybdenum exhibits exceptional mechanical and thermal behavior due to its ductility [1]. Moreover, the excellent electrical conductivity, low coefficient of thermal expansion, and high-temperature stability make it suitable for extreme condition applications, such as in the rocket motors, satellite antennae, laser optics, and nanometer-scale electronics [2,3,4]. The electrical conductivity and passive intermodulation (PIM) products which may cause serious interference problems in antenna applications [5,6] can be greatly improved after gold plating. However, the gold coating could be contaminated or damaged in the fabrication process or during use in space atmosphere. Moreover, the poor stability of contact resistance is considered to be one of the major causes of observed PIM effects [7,8]. In the presence of the above described situations, a polymer coating treatment on the surface of gilt molybdenum wire can be a promising and efficient approach to solve these problems.

Polyimide (PI) is a polymer with a highly aromatic structure which imparts it excellent thermal stability, unique electrical properties, superior chemical and radiation resistance, together with relatively good mechanical strength [9,10,11,12]. Compared to the flaws of the polymers which have already been widely used in space, such as the poor heat resistance of polyester or the inferior radiation resistance of Kevlar, these advantages make PI an ideal candidate polymer material for aerospace applications. Many such applications, like PI insulation foam [13], flexible film solar cells [14], film antenna substrate [15], have been explored. However, little has been reported about the application of combining PI with metallic materials which can also widely be used in the outer space environment [16]. So far, a lot of coating methods have been invented to coat PI onto the surface of other materials. Kim et al. [17] obtained an ultrathin PI-coated Li cathode which displayed an advanced electrochemical performance by a modified dip-coating method, but the ultrathin coating layer was observed to be heterogeneous. Naganuma et al. [18] applied a high-temperature vapor deposition method to prepare PI coating for the elimination of surface flaws on carbon fibers. Nevertheless, this method is too complex and costly for enabling large-scale production capabilities. Interestingly, Yuan et al. [19] used a conventional sizing treatment approach to coat PI membranes on carbon fibers, which was manifested to be relatively efficient.

In order to protect the gilt surface against contamination and mechanical damage and improve the electrical performance when employed as knitted meshes in large deployable antennae, in this study, a PI coating with high-temperature and chemical resistance was prepared onto the ultrafine gilt molybdenum wire by a modified sizing treatment and thermal imidization method. To the best of our knowledge, this is the first study on such a combination of PI coating and gilt molybdenum used for aerospace applications. The structural characteristics of the PI coating, the contact resistance, and microwave reflection performance of its warp knitted mesh were investigated. It was observed that this PI-coated gilt molybdenum wire possesses a great potential for aerospace applications.

## 2. Materials and Methods 

### 2.1. Materials

A gilt molybdenum wire with a diameter of 27 μm provided from Chengdu Hongbo Industrial Co., Ltd. (Chengdu, China) and Northwest Institute for Non-ferrous Metal Research (Xi’an, China) was used in this research. This gilt molybdenum wire was produced through special process, so its flexibility and tensile properties can meet the requirements of a further knitting process [20].

Pyromellitic dianhydride (PMDA, molecular weight: 218.12) and oxydianiline (ODA, molecular weight: 200.23) were supplied by the Shanghai Synthetic Resin Institute (Shanghai, China). *N*, *N*-dimethylacetamide (DMAc) was obtained from Shanghai Lingfeng Chemical Reagent Co., Ltd. (Shanghai, China) Acetone from Shanghai Tiancheng Chemical Co., Ltd. (Shanghai, China) was used a as dilution solvent in the coating process. All these chemicals had a purity of 99% and could be used without further purification.

### 2.2. Preparation of PAA Solution

As the precursor of PI, a Polyamic acid (PAA) solution was made by condensation polymerization of PMDA and ODA. An amount of 32.72 g (0.15 mol) of ODA was dissolved in 500 mL of DMAc in a flask under nitrogen purging at a temperature of −15 °C. Subsequently, after an intensive mixing, 30.03 g (0.15 mol) of ODA were added slowly into the flask. The solution was then stirred under nitrogen for 2 h at −15 °C, 1h at 0 °C, and an additional 1 h at 15 °C. Finally, after aging with stirring for 24 h at room temperature, and sonication for 1 h, a 25 wt % yellow-green PAA solution was obtained. To prepare the coating sizing, the previous solution was diluted with acetone at a volume ratio of 1:1.

### 2.3. Fabrication of PI-Coated Gilt Molybdenum Wire and Its Knitted Mesh

**Figure 1:**
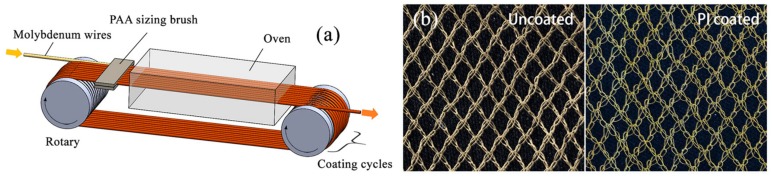
(**a**) Modified sizing treatment setup for polyimide (PI) coating on ultrafine wire; (**b**) samples of uncoated and PI-coated gilt molybdenum wire meshes.

A felt brush with a width of 40 mm and density of 0.16 g/cm^3^ was dipped in PAA sizing whose amount was controlled by a pump. Following the brush, an oven with a length of 3.93 m was used, with segments at the controlled temperatures of 100 °C, 200 °C, 300 °C, 420 °C, 300 °C, respectively. The length of each temperature zone was about 0.8 m. The uncoated gilt molybdenum wires passed in sequence through the PAA sizing brush and the oven, and looped for designed times with a speed of 25 m/min. Afterwards, PI coatings with a thickness of 3 μm, 4 μm, 5 μm were formed on the surface of the gilt molybdenum wires with sizing treatment cycles of 6, 8, and 10 times, respectively. The coating process is shown in Figure 1a.

Subsequently, the PI-coated gilt molybdenum wires were fabricated into mesh fabrics by a modified warp knitting machine designed for metal wires, which was discussed in our previous study [21]. An uncoated gilt molybdenum wire mesh was also prepared for comparative purposes, as shown in Figure 1b.

### 2.4. Fourier-Transform Infrared Spectroscopy (FTIR)

Infrared spectra of the samples were recorded using a Fourier-transform infrared (FTIR) spectrometer (Nicolet 6700, Thermo Fisher Scientific Inc., Waltham, MA, USA) in attenuated total reflection (ATR) mode. To confirm that the chemical composition of the coated film was PI, FTIR spectra of the coated gilt molybdenum wire after thermal imidization were obtained at a resolution of 4 cm^−1^ in the range of 400–4000 cm^−1^ for 10 times. The spectra were then analyzed by OMNIC 8.2 software (Thermo Fisher Scientific Inc., Waltham, MA, USA).

### 2.5. Morphology Study

The surface morphology images were obtained by using a scanning electron microscope (SEM, JSM-5600LV, JEOL, Tokyo, Japan) with an accelerating voltage of 15 kV. To confirm that the coated film had successfully formed and attached, the elements on the surface of the uncoated and coated gilt molybdenum wires were measured by an Energy-Dispersive Spectrometer (EDS, IE300X, Oxford Instruments, Abingdon, UK) with an accelerating voltage of 20 kV. A superlow-temperature treatment of the PI-coated gilt molybdenum wires was performed in liquid nitrogen at −196 °C for 24 h, and a high-temperature treatment was carried out in a vacuum oven at 300 °C for 24 h, after which the surface morphology was observed by SEM to examine the stability at extreme temperatures.

### 2.6. Tensile Properties

The tensile tests of the coated and uncoated gilt molybdenum wires were performed by a yarn tensile testing machine (YG061-1KG, Laizhou Electron Instrument Co., Ltd., Shandong, China) with a cross-head speed of 100 mm/min. At least 10 specimens of each type were tested. Additionally, the tensile behavior of the PI-coated and uncoated gilt molybdenum wire mesh fabrics was tested with a cross-head speed of 100 mm/min on a universal tensile tester (WDW-20, Shanghai Hualong Test Instrument Co., Ltd., Shanghai, China).

### 2.7. Wire Mesh Contact Resistance

**Figure 2:**
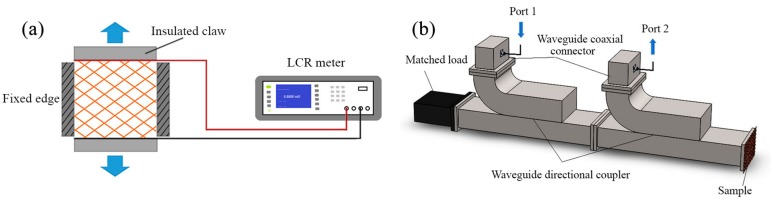
Schematic illustrations of the test setups. (**a**) Scheme of the contact resistance stability test and (**b**) the microwave reflection performance test used for the wire meshes.

Normally, the electrical performance of the wire mesh fabric will change when experiencing small deformations. To examine the stability of the electrical performance under real conditions which involve tension and sometimes small movements, an experimental device with a stretching machine was built, as shown in Figure 2a. In this paper, we used resistance measured by an inductance, capacitance and resistance (LCR) meter (DM3068, Beijing RIGOL Technology Co., Ltd., Beijing, China) to characterize the electrical performance. The tensions were generated by an X-Y biaxial tensile tester (Wenzhou Darong Textile Instrument Co., Ltd., Wenzhou, China) with four insulating claws. In the test, two X-direction claws were holding the fixed edge of the samples with tension, while two Y-direction claws were moving at a slow speed of 10 mm/min until reaching a strain of 10%. Meanwhile, the resistance change between the two ends of the mesh fabrics during this process was measured and recorded by an LCR meter. The relative change in resistance *ΔR*/*R*_0_ was then calculated, where *ΔR* is the real-time resistance change, and *R*_0_ is the initial resistance.

### 2.8. Microwave Reflection Properties

In this work, the waveguide reflection method was employed to dielectrically characterize the electromagnetic microwave reflection properties of the PI-coated and uncoated gilt molybdenum wire mesh fabrics. Thus, an S-parameter measurement system was prepared. The placement of the test components in this system is shown in Figure 2b. S-band waveguides WR430 (Xi’an microwave telecom technology Co., Ltd., Xi’an, China) working in the frequency bands 1.7–2.6 GHz were used, where the mesh samples and a properly matched microwave-absorbing load were fixed at the two ends, respectively. The microwave scattering parameter S21 was measured by an Agilent-N5230A network analyzer (Agilent Technologies Inc., Santa Clara, CA, USA) which was connected to coaxial connectors through Port1 and Port2 in a frequency range of 1.8–2.4 GHz. Before testing, a metal plate was used as a standard sample to calibrate the measurement, getting a reflection effectiveness of 0 dB. The S-parameters were obtained and analyzed later, using a computer. Based on the average magnitude and phase of S21 of five marker points (1.9 GHz, 2.0 GHz, 2.1 GHz, 2.2 GHz, and 2.3 GHz), the reflectivity of those specimens could be calculated according to the relationship between return loss and reflectivity:

(1) $$RL=-10\log_{10}RF$$

where *RF* is the reflectivity, and *RL* is the average return loss at the marked frequencies.

## 3. Results and Discussion

### 3.1. FTIR Analysis

**Figure 3:**
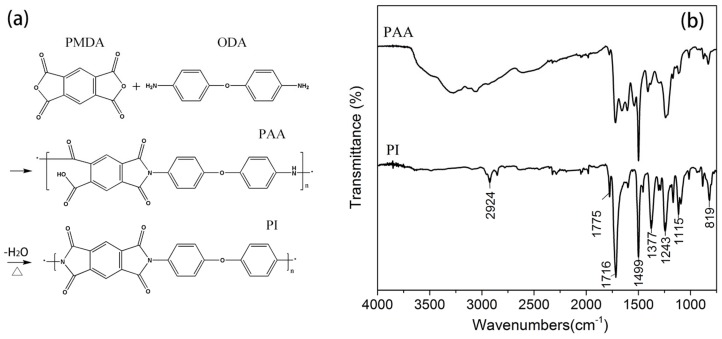
(**a**) Polymerization of PI by reaction between the oxydianiline (ODA) and pyromellitic dianhydride (PMDA) precursors; (**b**) FTIR spectra of PAA sizing and PI coating on the gilt molybdenum wire surface.

After coated and heated on the coating device, the PAA should have converted into PI. The FTIR spectra of the coated gilt molybdenum wire is shown in Figure 3. The absorption bands observed at 1775 and 1716 cm^−1^ were attributed to the asymmetric and symmetric stretching of C=O in the imide ring, respectively. The C–N stretching vibration at 1377 cm^−1^ belonged to the characteristic peaks of the amide groups [22,23]. The absorption bands at 2924 and 1499 cm^−1^ were generated by stretching of C–H and C=C in the benzene ring, respectively, whereas the band at 819 cm^−1^ was due to the symmetrical elongation of the aromatic nucleus. The characteristic peaks of C–O–C stretching were observed at 1243 and 1115 cm^−1^. This spectrum displayed the typical pattern of PI and indicated the imidization of PAA sizing to PI coating. Thus, it was anticipated that the original gilt molybdenum wire was significantly coated by PI.

### 3.2. Surface Morphology and Temperature Stability

**Figure 4:**
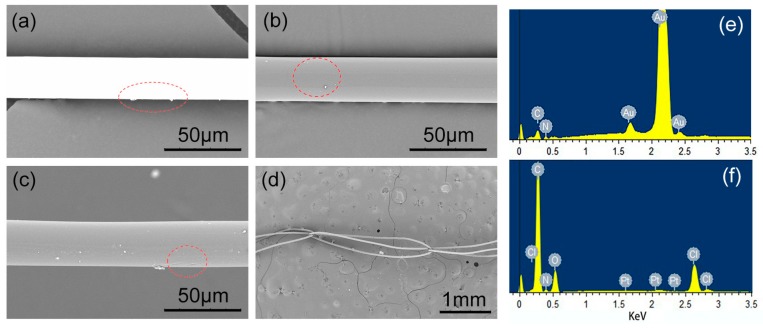
SEM images of (**a**) uncoated and (**b**) PI-coated gilt molybdenum wires. PI coating morphology (**c**) after temperature treatment and (**d**) after warp knitting process. Energy-Dispersive Spectrometry (EDS) patterns of (**e**) uncoated and (**f**) coated gilt molybdenum wires.

SEM images of the uncoated and PI-coated gilt molybdenum wires are shown in Figure 4. It can be seen in Figure 4a,b that the surface of the gilt molybdenum wires changed uniformly from bright white to dark grey after coating, which indicated that a continuous and uniform PI coating had been obtained on its surface. However, compared to PI-coated copper wires, some tiny particles were observed on the surface of the PI-coated gilt molybdenum wires. This may have been caused by granular molybdenum from the gilt molybdenum wires [24].

A more detailed image of the surface elements before and after coating can be seen in the pattern of EDS in Figure 4. The main ingredient of the gilt molybdenum wires surface before coating was Au, accounting for 93.23 wt %, as shown in Figure 4e. However, the spectrum peaks in Figure 4f showed that, after coating, the surface was mainly composed of C, N and O elements which indicated that an insulating polymer film had successfully coated the surface of the gilt molybdenum wires. This result was in accordance with the FTIR analysis and SEM images.

As mentioned before, PI-coated gilt molybdenum wires were aimed to be used in extreme conditions, such as space environment. The majority of space missions have been flown in the Earth’s orbit where extreme temperatures can be reached, typically in the range of −190 and 160 °C [1]. Hence, evaluations of extreme temperature stability for potential applications of the PI-coated gilt molybdenum wires were required. In Figure 4c, PI coating with a thickness of 3 μm on the surface of gilt molybdenum wires did not show a substantial morphological variation but only small wrinkles after immersion in liquid nitrogen at −196 °C for 24 h and heating at 300 °C for 24 h. Since PI contains heteroaromatic units which show high heat resistances and high glass transition temperatures, it is stable up to a temperature of 440 °C without oxidation [9,11]. Hence, the PI-coated gilt molybdenum wires presented an excellent performance for extreme condition applications.

### 3.3. Tensile and Wear Performance

**Figure 5:**
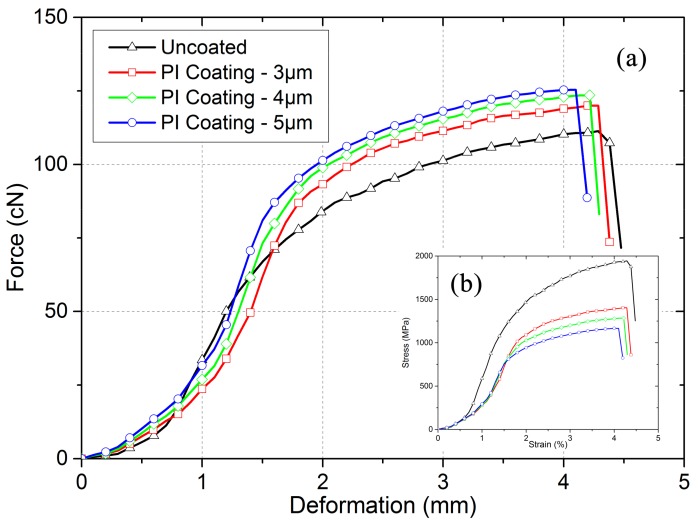
Tensile behavior of uncoated and PI-coated gilt molybdenum wires. (**a**) Force change of the wires in response to deformation; (**b**) stress–strain relationships during stretching.

The tensile results of various gilt molybdenum wires are presented in Figure 5. In PI-coated gilt molybdenum wires, the molybdenum wires undertook most of the load, and the composite wires failed when the molybdenum wires broke. This resulted in the curves being similar. Before the ultimate tensile stress, the curves kept rising with a mode shaped like an “S”, with the beginning showing a small linear deformation area. After the ultimate tensile stress, the curves dropped sharply, which indicated the complete breakage of the composite wires. Compared to the uncoated gilt molybdenum wires, the PI-coated gilt molybdenum wires presented a higher broken force. The main reason for this was likely that the coating could help to heal the flaws and cracks on the gilt molybdenum wires to some extent [18,19,25]. Moreover, we can see from Figure 5b that the initial moduli of the PI-coated gilt molybdenum wires were similar to those of the uncoated wires. The tensile properties of the PI-coated gilt molybdenum wires with different coating thicknesses of 3 μm, 4 μm, and 5 μm are also discussed. With the increase of the coating thickness of PI, the composite wires exhibited higher broken forces but a similar initial modulus.

The wear performance of the PI coating membrane was examined by a further knitting process. During the process, the wires were rubbed against the needles several times, after which the surface morphology was observed via SEM, as shown in Figure 4d. The image indicated that the interfacial bonding of the composite wire was strong enough for the fabrication process, and the coating played an efficient role in protecting the surface of the gilt molybdenum.

### 3.4. Mesh Contact Resistance Stability

In some potential working environment, the tension variation of the wire mesh induced by a temperature change or by an environmental vibration would affect the contact conditions of the wires and result in unstable electrical properties of the wire mesh.

**Figure 6:**
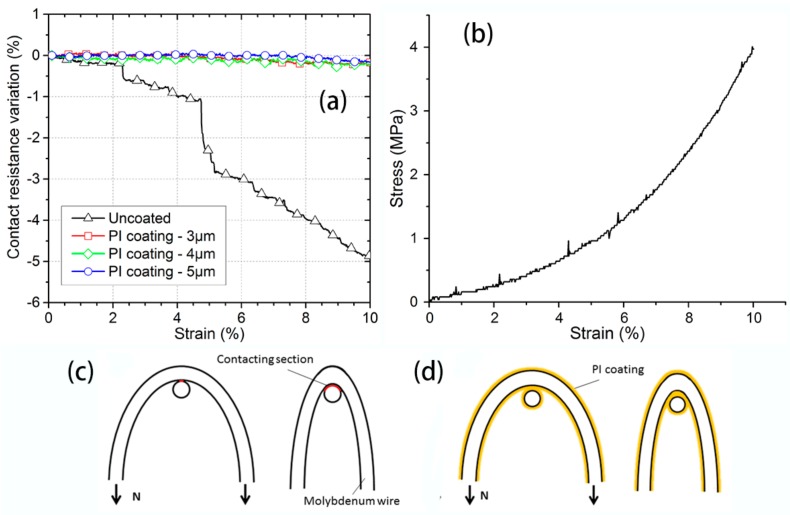
(**a**) Mesh contact resistance of uncoated and PI-coated gilt molybdenum wire meshes during stretching. (**b**) A typical stretching curve of wire meshes. Change of the contact sections of (**c**) uncoated and (**d**) PI-coated gilt molybdenum wire meshes under increasing loads.

Figure 6a shows the mesh contact resistance variation of the uncoated and PI-coated gilt molybdenum wire mesh under a small strain, below 10%. The results indicated that the resistance of the uncoated gilt molybdenum wire mesh was very sensitive to tension. It can be seen that the mesh resistance kept reducing with the increase of strain. As we assumed that the jamming of wires did not occur, the circuit topology would not change during the deformation process of the fabric when under a small strain. Therefore, the resistance variation was greatly governed by the contact resistance which changed according to the applied tensile force [26]. With the increase of load, the contact area would get larger as illustrated in Figure 6c, thereby decreasing the contacting resistance. Several sharp drops were observed in the resistance variation curve. These resistance changes could be attributed to the wires slipping in the knitted structure during stretching, which was in accordance with the tensile behavior of the knitted mesh fabric shown in Figure 6b. This is due to the fact that after moving and slipping, the contact points of the wires increased, and some loose contacts also improved. The contact resistance stability of the PI-coated gilt molybdenum wire meshes with different coating thicknesses in the increasing value of 3 μm, 4 μm, and 5 μm are also displayed in Figure 6a. It was observed that the three resistance variation curves lay very close to each other and maintained a relatively stable value, as the PI coating membrane had isolated the contact of the conductive wires, as shown in Figure 6d, so that the contacting resistance would not change under small strain. Thus, PI coating could efficiently enhance mesh contact resistance stability and further improve the electrical stability.

### 3.5. Electromagnetic Microwave Reflection Properties

**Figure 7:**
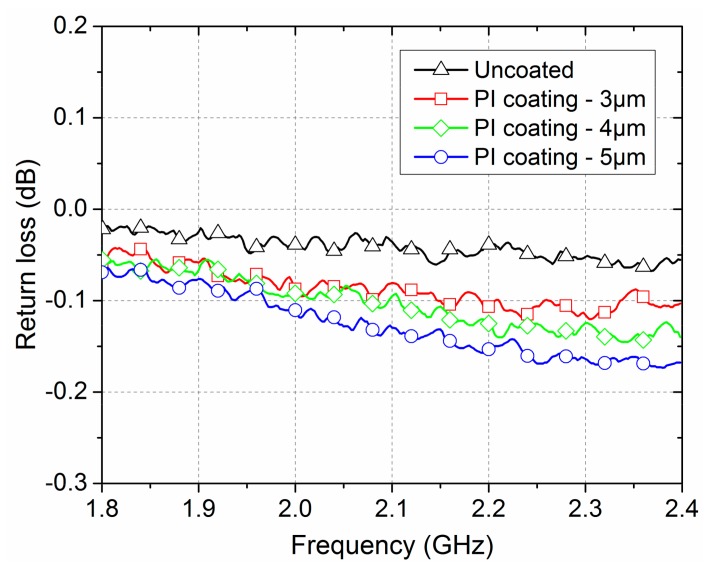
Mesh contact resistance of uncoated and PI-coated gilt molybdenum wire meshes during stretching.

Figure 7 illustrates the return loss spectra of the PI-coated and uncoated gilt molybdenum wire meshes as a function of frequency in the range of 1.8–2.4 GHz. It could be seen that the return loss values of the PI-coated and uncoated gilt molybdenum wire meshes had similar change trends and gradually increased with the increase of the frequency. As the wavelength of the electromagnetic microwave became shorter with the increase of the frequency, a higher penetrability was possible [27,28]. Furthermore, the uncoated gilt molybdenum wire mesh samples showed a lower return loss than the PI-coated gilt molybdenum wire mesh samples. The electromagnetic microwave reflectivity of the uncoated and of the 3 μm PI-coated gilt molybdenum wire mesh were 99.5% and 98.1%, respectively. The reason may be the dielectric loss caused by the coating membrane. According to our previous research, there were some molybdenum particles both on and in the raw gilt molybdenum wires [21]. After coating, a part of these conductive molybdenum particles dispersed in the PI coating membrane acting as absorbents. Subsequently, the main loss mechanisms of such composites could be explained by the relaxation polarization loss and the electric conductance loss [29,30,31]. The thickness of the insulated coating membrane enlarged the gap between the conductive wires, which may also lead to the increase of the electromagnetic transmission. As can be seen in Figure 7, the return loss of the PI-coated gilt molybdenum wire mesh increased with the increase of the coating thickness from 3 μm to 5 μm. The reflectivity of the PI-coated gilt molybdenum wire meshes with thickness of 3 μm, 4 μm, and 5 μm were 98.1%, 97.7% and 97.1%, respectively.

In general, the PI coating membrane would affect the electromagnetic microwave reflection properties. Nevertheless, with a moderate coating thickness, the PI-coated gilt molybdenum wire mesh could still retain a reasonable electromagnetic microwave reflection performance.

## 4. Conclusions

Polyimide-coated gilt molybdenum wires and their knitted mesh fabrics were successfully designed and fabricated. Experimental results demonstrated that a PI membrane could improve the tensile properties of gilt molybdenum wire and play an important role in protecting the gilt during knitting process. Moreover, it had the ability to resist the extreme temperatures from −196 °C to 300 °C. This kind of PI-coated gilt molybdenum wire mesh exhibited excellent contact resistance stability when under a small tension. As the thickness of the PI coating membrane increased, the electromagnetic microwave reflectivity of the wire mesh decreased. With a moderate coating thickness of 3 μm, the reflectivity could still remain at 98.1%, which was above the value required by antenna applications.
